# Optimization Study of the Capacity of *Chlorella vulgaris* as a Potential Bio-Remediator for the Bio-Adsorption of Arsenic (III) from Aquatic Environments

**DOI:** 10.3390/toxics11050439

**Published:** 2023-05-06

**Authors:** Reem Mohammed Alharbi, Essam Nageh Sholkamy, Khawla Ibrahim Alsamhary, Neveen Abdel-Raouf, Ibraheem Borie M. Ibraheem

**Affiliations:** 1 Department of Biology, College of Science, University of Hafr Al Batin, Hafr Al Batin 39524, Saudi Arabia; 2 Department of Botany and Microbiology, College of Science, King Saud University, Riyadh 11451, Saudi Arabia; 3 Department of Biology, College of Science and Humanities in Al-Kharj, Prince Sattam Bin Abdulaziz University, Al-Kharj 11942, Saudi Arabia; 4 Botany and Microbiology Department, Faculty of Science, Beni-Suef University, Salah Salem Street, Beni-Suef 62511, Egypt

**Keywords:** *Chlorella vulgaris*, arsenic (III), Fourier-transform infrared spectrophotometer (FTIR), Langmuir, Freundlich, Dubinin–Radushkevich, bio-adsorption

## Abstract

This study examined the ability of the green microalgae *Chlorella vulgaris* to remove arsenic from aqueous solutions. A series of studies was conducted to determine the optimal conditions for biological arsenic elimination, including biomass amount, incubation time, initial arsenic level, and pH values. At 76 min, pH 6, 50 mgL^−1^ metal concentration, and 1 gL^−1^ bio-adsorbent dosage, the maximum removal of arsenic from an aqueous solution was 93%. The uptake of As (III) ions by *C. vulgaris* reached an equilibrium at 76 min of bio-adsorption. The maximum adsorptive rate of arsenic (III) by *C. vulgaris* was 55 mg/gm. The Langmuir, Freundlich, and Dubinin–Radushkevich equations were used to fit the experimental data. The best theoretical isotherm of Langmuir, Freundlich, or/and Dubinin–Radushkevich for arsenic bio-adsorption by *Chlorella vulgaris* was determined. To choose the best theoretical isotherm, the coefficient of correlation was used. The data on absorption appeared to be linearly consistent with the Langmuir (q_max_ = 45 mgg^−1^; R^2^ = 0.9894), Freundlich (k_f_ = 1.44; R^2^ = 0.7227), and Dubinin–Radushkevich (q_D–R_ = 8.7 mg/g; R^2^ = 0.951) isotherms. The Langmuir and Dubinin–Radushkevich isotherms were both good two-parameter isotherms. In general, Langmuir was demonstrated to be the most accurate model for As (III) bio-adsorption on the bio-adsorbent. Maximum bio-adsorption values and a good correlation coefficient were observed for the first-order kinetic model, indicating that it was the best fitting model and significant in describing the arsenic (III) adsorption process. SEM micrographs of treated and untreated algal cells revealed that ions adsorbed on the algal cell’s surface. A Fourier-transform infrared spectrophotometer (FTIR) was used to analyze the functional groups in algal cells, such as the carboxyl group, hydroxyl, amines, and amides, which aided in the bio-adsorption process. Thus, *C. vulgaris* has great potential and can be found in eco-friendly biomaterials capable of adsorbing arsenic contaminants from water sources.

## 1. Introduction

Heavy metals are found randomly and naturally in the layers of the Earth and water sources, but they negatively affect human life if they exceed internationally permissible concentrations [1,2]. It is typical for living creatures on Earth to inhale these pollutants and thus influence the general health of these neighborhoods [3,4,5]. A population explosion worldwide has led to the increase and development of more industries, resulting in an increased release of pollutants into the environment [5,6,7]. One type of pollution is the direct or indirect release of waste materials or energy into water, air, or soil, which causes short-term or chronic long-term alterations to the global ecological balance, lowering the quality of natural life on Earth [8]. Bio-adsorption materials, especially algae, are abundant in nature either in terrestrial, marine, or freshwater ecosystems, and can also be cultivated under optimized conditions [8]. Hence, the environment has been searching for an inexpensive and efficient technology for the treatment of metal-containing waste materials. The use of biological materials to remove metals is one such technology that has received considerable attention in the past two decades.

Arsenic is a naturally occurring element in the Earth’s crust and is widely distributed throughout the environment, including in water, air, and soil. It is exceedingly poisonous in its inorganic form and is one of ten compounds classified by the World Health Organization as a significant public health concern [9]. The current standard for arsenic in drinking water is 10 micrograms per liter. Drinking polluted water, using that water in food preparation, irrigating food crops, industrial processing, eating contaminated foods, and smoking tobacco all expose a person to high levels of inorganic arsenic in the body. Arsenic is used as an alloying agent in manufacturing glass, dyes, textiles, paper, metal adhesives, wood preservatives, and ammunition [10]. One of the strongest and most hazardous heavy metals is arsenic [11]. Water-soluble arsenic trioxide, which is slowly absorbed via the intestine and has a lethal dose of between 60 and 20 milligrams, is one of the most dangerous poisonous arsenic compounds [12]. The hazardous effects of arsenic trioxide start to show after a quarter-hour to several hours [13]. Another type of arsenic is arsenic gas, which is absorbed directly into the bloodstream through inhalation. Even minute levels in the surrounding air can induce significant poisoning through blood-cell disintegration [14]. The gas is created by employing acids to purify minerals polluted with arsenic impurities [10]. Therefore, some of the general methods that are used for arsenic adsorption include chemical oxidation, iron-sheathed sand and gravel, adsorption, ion-exchange resin, activated alumina, membrane processes, and nano-filtration. Although some of these advanced methods are frequently expensive [11], adsorption is a cost-effective, simple, and efficient method for the adsorption of heavy metal ions from waste [10].

Heavy metals are not biodegradable because they do not decompose or deteriorate when they penetrate the cells of living organisms [8,15,16]. Toxin accumulation in the food chain necessitates the development of long-term methods to eliminate them efficiently. Traditional approaches for this include nano-processing, sophisticated oxidation procedures, electrodeposition, electrocoagulation, membrane separation, the use of solvents, and ion exchange, all of which are expensive and have substantial downsides [17,18]. Some examples are a low transmission, a high cost, insufficient removal, excessive energy consumption, or a highly harmful waste output [19]. Modern science has turned to other methods such as biological treatments, which are simple, low-cost methods based on the employment of microorganisms [20,21]. A natural treatment produces less effluent than conventional methods because biological approaches are a promising alternative and prospective solution to overcome the limitations of previous procedures [22]. Heavy metals are biologically remediated through bio-accumulation and natural adsorption from aqueous environments by passive binding to dry biomass [23]. Many microorganisms, including microscopic algae, are exploited in this approach [24]. The usage of algae is regarded as the finest biological method for heavy metal treatments because of the sugars, proteins, and lipids found on the surface of their cell walls, which easily attach to these minerals, as well as their cell wall contents [25,26]. They include active groups that act as binding metals such as hydroxyl, carboxyl, amine, and sulfate [27]. A recent study also demonstrated the ability of two types of microalgae, *Phormidium tenue* and *C. vulgaris*, as bio-sorbent materials to remove them from aqueous solutions [23].

Algae create more biomass than other microbes and, for the most part, do not produce hazardous chemicals [28]. *C. vulgaris* has lately gained popularity for treating heavy metal pollution in aquatic environments [2,29]. The present study examined the feasibility of using the green algae *C. vulgaris*, which was employed in this study to observe its ability to adsorb arsenic from an aqueous external environment.

## 2. Materials and Methods

### 2.1. Sodium Arsenite

The stock solution of NaAsO_2_ was made at a concentration of 1000 mgL^−1^ as the source of arsenic (III). After that, the solutions were diluted to the desired concentration and tested [30]. All chemicals and reagents used were purchased from Merck (Fischer Chemicals, Zurich, Switzerland).

### 2.2. Microalgal Sample

*Chlorella vulgaris* (SAG 211-11b) was the name of the microalgal strain employed in this study. This strain was purchased from Goettingen University’s Culture Collection of Algae and re-cultured in 10 mL of *Chlorella vulgaris*-appropriate Fog’s Medium.

### 2.3. Preparation of Adsorbent Biomass

These steps were used to prepare the microalgal biomass. First, the algal biomass was deactivated in an autoclave at 121 °C for 10 min. Second, the deactivated biomass was collected by filtering cultured media using cellulose nitrate Whatman membranes with pore sizes of 45 m and washed with deionized water. Third, the biomass was sun-dried for three days before being oven-dried at 55 °C for 24 h and pulverized in a stone mortar and pestle to achieve a uniform particle size. For future usage, forty sieved materials were stored in an airtight container [31].

### 2.4. Factors Affecting the Adsorption Process

The optimal conditions for arsenic adsorption by *C. vulgaris* were determined in batch mode at 30 ± 1 °C using several parameters such as pH (2, 4, 6, and 8), biomass dose (0.25, 0.5, 0.75, 1.0, 1.25, 1.5, 1.75, and 2.0 gL^−1^), contact interval (15, 30, 60, 120, 150, and 180 min), and initial concentration (25, 50, 75, 100, 150, and 200 mgL^−1^). Experiments were carried out in three copies in a 150 rpm orbital incubator using 250 mL conical flasks containing 100 mL of arsenic solution. After centrifugation incubation at 4000 rpm for 10 min, the microalgal biomass was separated. The supernatant was filtered via 0.45-micron Millipore filters, and the filtering was tested for residual arsenic using an atomic absorption spectrometer set at 283.3 nm (VARIAN, Inc., AA24OFS model).

### 2.5. Elemental Analysis

The chemical constituents of the algal adsorbent (% of carbon, hydrogen, nitrogen, and oxygen) were analyzed using an elemental analyzer (Eltra GmbH, Retsch-Allee, Haan, Germany).

### 2.6. FTIR Analysis

The functional groups of the algal adsorbent and arsenic (III)-loaded algal adsorbent were further investigated using a Fourier-transform infrared (FTIR) spectrophotometer (Mattson Satellite 5000 FTIR, Warwickshire, UK) in the 400–4000 cm^−1^ wave number range.

### 2.7. Morphological Study of Adsorbent Surface

The untreated and arsenic-treated adsorbents were collected and prepared for the SEM (Zeiss, Sigma VP, Jena, Germany) examination [32].

### 2.8. Adsorption Isotherm

The optimum concentration of chemical reagents (1 M NaCl for *C. vulgaris*) was utilized in the adsorption isotherm analysis under conditions such as an adsorbent dosage of 1 g, varied initial arsenic (III) concentrations (10 to 300 mgL^−1^), and agitation at 150 rpm and 25 °C for 8 h to ensure that the adsorption system attained an equilibrium, with the initial solution pH adjusted to 6.0.

The amount of equilibrium adsorption (qe mgg^−1^) of the As (III) was calculated following Equation (1):

(1) $$q_{e}=\frac{V\times (C_{0}-C_{e})}{W}$$

where V (L) denotes the solution volume, W (g) represents the dry adsorbent weight, and C_0_ (mgL^−1^) represents the initial As (III) concentration. C_e_ (mgL^−1^) represents the (III) concentration at the equilibrium.

The Langmuir and Freundlich models [33] in Equations (2)–(5), respectively, and the Dubinin–Radushkevich (D–R) isotherms in Equations (6) and (7) were employed to evaluate the adsorption isotherms to assess the performance of the adsorbents.

(2) $$q_{e}=\frac{q_{\max}K_{L}C_{e}}{1+K_{L}C_{e}}.$$

(3) $$\frac{C_{e}}{q_{e}}=\frac{1}{q_{\max}}+\frac{C_{e}}{q_{\max}}$$

(4) $$q_{e}=K_{F}{\left(C_{e}\right)}^{1/n}$$

(5) $$\mathrm{In}\;q_{e}=\frac{1}{n\;\mathrm{In}\;C_{e}}+\mathrm{In}K_{F}$$

(6) $$E^{2}\mathrm{In}\;qe=\mathrm{In}\;k_{D–R}-\beta$$

(7) $$E=\frac{1}{\sqrt{2\beta^{2}}}$$

where q_max_ (mgg^−1^) represents the maximum adsorption capacity, q_e_ (mgg^−1^) represents the equilibrium-adsorbed amount of As (III), and C_e _(mgL^−1^) denotes the solute’s equilibrium concentration. The Langmuir constant for adsorption energy is represented by K_L_, whereas K_F_ is the magnitude of bio-adsorption capacity; n is the bio-adsorption intensity, K_D–R_ and β are D–R constants, and E is energy for the uptake of arsenic on the bio-adsorbate surface.

### 2.9. Bio-Adsorption Kinetics

The bio-adsorption kinetics were investigated in 50 mL of solution under conditions such as 30 mgL^−1^ As (III), 1 gL^−1^ adsorbent, pH 6, and agitation at a speed of 150 rpm and a temperature of 25 °C, with time intervals ranging from 5 min to 8 h, depending on the appropriate equilibrium time. The adsorption kinetic data on the removal of As (III) were assessed via pseudo-first- and second-order models. Equation (8) expresses the pseudo-first-order kinetic model.

(8) $$\ln(q_{e}-q_{t})=\ln q_{e}-k_{1}t$$

Equation (9) shows the pseudo-second-order kinetic model:

(9) $$\frac{t}{q_{t}}=\frac{1}{k_{2}q^{2}}+\frac{t}{q_{e}}$$

where q_e_ and q_t_ represent the equilibrium adsorption capacity (mgg^−1^) and the adsorption capacity at t (min), respectively, and k_1_ and k_2_ are the rate constants for the pseudo-first-order and pseudo-second-order models, respectively. All the experiments were duplicated and the averages were used for the analysis.

### 2.10. Thermodynamics of As (III) Bio-Adsorption

The uptake of arsenate ions by *Chlorella vulgaris* was investigated by varying the temperature from 10 to 50 °C at optimum conditions and evaluating the temperature effect using the following equations:

(10) In=kc-ΔH/RT+ΔS/R

(11) ΔG=-RT lnKc

where Kc is the equilibrium constant; ΔH, ΔS, and ΔG are the standard enthalpy, entropy, and Gibbs free energy, respectively; T is the temperature (kelvin); and R is the gas constant.

## 3. Results and Discussion

### 3.1. Optimization of the Bio-Adsorption Conditions

#### 3.1.1. Arsenic Bio-Adsorption at Different pH Values

**Figure 1:**
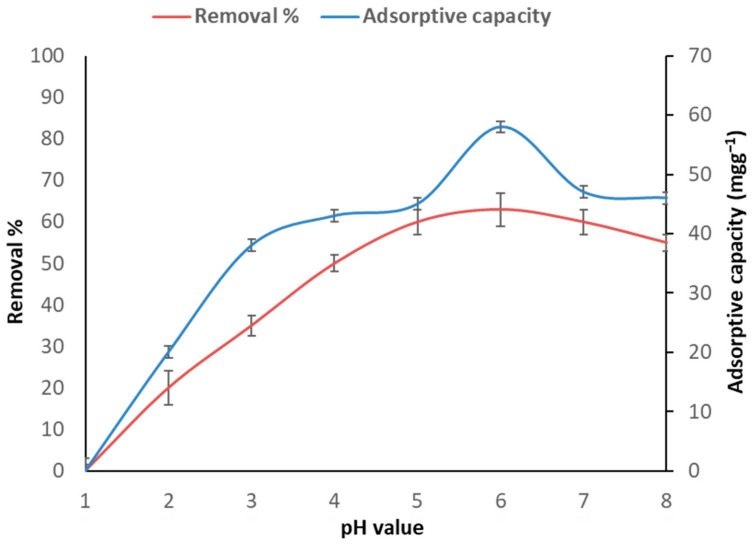
Bio-adsorption capability of *C. vulgaris* adsorbent at various pH values, 100 gL^−1^ arsenic concentration, 1 g adsorbent, and 120 min.

The bio-adsorption capability was highly dependent on pH, as shown in Figure 1. The optimal pH for adsorption at 100 gL^−1^ arsenic, 1 g bio-adsorbent, and a 120 min incubation was determined using a pH range of 1–8. The bio-adsorption rate was limited at an initial pH of 3.0 and reached a maximum at an optimal pH of 6.0, with a bio-adsorption rate of 58 mgg^−1^; the removal percentage was 63%. According to Abdel-Raouf and colleagues, determining the optimal pH has a significant impact on regulating the metal aqueous and functional groups of adsorbent surfaces, thereby influencing heavy metal adsorption in an aqueous environment [34]. Bio-adsorption has been discovered to be relatively stable above a pH of 6.0 [35]. Due to the high concentration of H^+^ hydrogen ions, the adsorption capacity diminishes when the pH falls below 3. In this instance, the rate of arsenic ions in the solution was lower than the concentration of H^+^ ions, increasing the possibility of competition with them for surface binding sites of adsorption due to repulsive forces between metal ions and proteins [36]. The absorbent material’s surface was fully covered by hydrogen ions at low pH values, thus preventing the AS (III) ions from binding to the adsorption sites. Additionally, the functional groups on the absorbent’s surface were influenced by the pH. Surface charge changes on these functional groups played a crucial role in determining the activity of the absorbent material. The increased positive charge (proton) density on the functional groups at low pH values created a barrier that prevented the AS (III) ions’ approach due to repulsive forces. Nonetheless, as the pH level increased, the competition from hydrogen ions decreased, with a greater proportion of absorbent functional groups becoming negatively charged. This led to a decrease in electromagnetic repulsion, facilitating the attraction of metal cations and adsorption on the absorbent material’s surface.

#### 3.1.2. As (III) Bio-Adsorption on Algal Adsorbent at Various Contact Times

**Figure 2:**
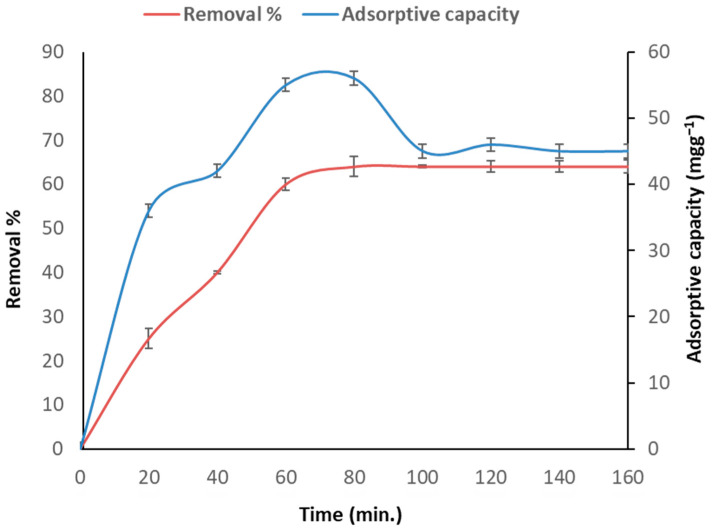
As (III) bio-adsorption rate by *C. vulgaris* adsorbent at various contact times, pH 6, 100 gL^−1^ arsenic concentration, and 1 g adsorbent.

Figure 2 shows the bio-adsorptive capacity and removal percentage of As (III) onto the surface of *C. vulgaris* at various contact times (20, 40, 60, 80, 100, 120, 140, and 160 min). After 76 min of bio-adsorption, *C. vulgaris*’ uptake of As (III) ions reached an equilibrium. The time it takes for heavy metals in a solution to reach a constant value is known as the adsorption equilibrium time. The maximum bio-adsorption rate for As (III) uptake was 55 mgg^−1^ and the removal percentage was 64%. According to a similar study, a rapid absorption rate was followed by a slower rate and, eventually, an equilibrium [37]. The rate of metal removal was initially rapid due to the abundance of binding sites on the biomass surface, which eventually became saturated. The rapid uptake of As (III) by the biomass binding sites demonstrated an adsorption process with no energy-mediated reactions occurring via surface binding. Due to the rapid metal bio-adsorption, the application was simplified and more efficient.

#### 3.1.3. Bio-Adsorption Capacity at Varying Bio-Adsorbent Doses

**Figure 3:**
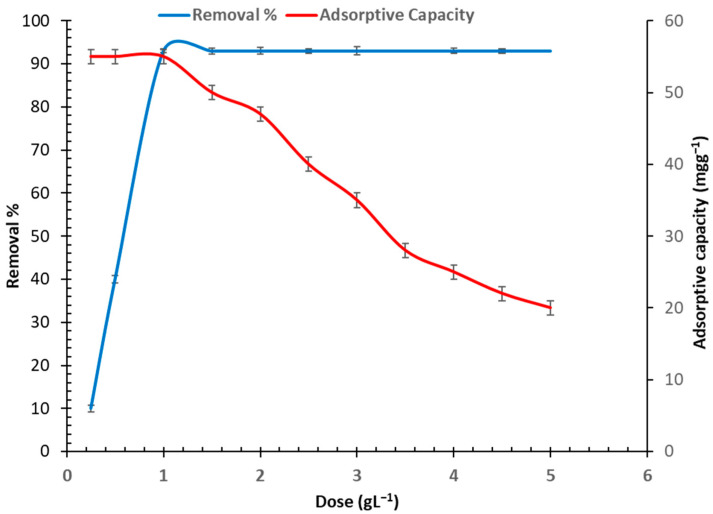
Determination of optimal algal adsorbent doses for arsenic adsorption by *C. vulgaris*. The initial concentration was 100 mgL^−1^, the pH was 6.0, and the contact time was 76 min.

The results depicted in Figure 3 show that increasing the bio-adsorbent dose from 0.25 to 1 gL^−1^ led to a rise in the As (III) ion removal percentage, from 8 to 100%. The removal percentage remained unchanged after the concentration reached 1 gL^−1^. In contrast, increasing the bio-adsorbent dosage between 1 and 5 mgg^−1^ reduced the bio-adsorption rate from 55 to 20 mgg^−1^. Consequently, the optimal bio-adsorbent dose was found to be 1 gL^−1^, with the highest rate of arsenic bio-adsorption by *C. vulgaris* being 55 mgg^−1^ and removal percentage of 93%. It should be noted that the adsorption rate decreased as the adsorbent dose increased due to the initial metal ion concentration being insufficient to match all the active sites on the adsorbent’s surface. This was an indicator of the attraction between the functional groups present on the adsorbent’s surface and the arsenic particles [38,39].

#### 3.1.4. Bio-Adsorption of As (III) at Different Metal Concentrations

**Figure 4:**
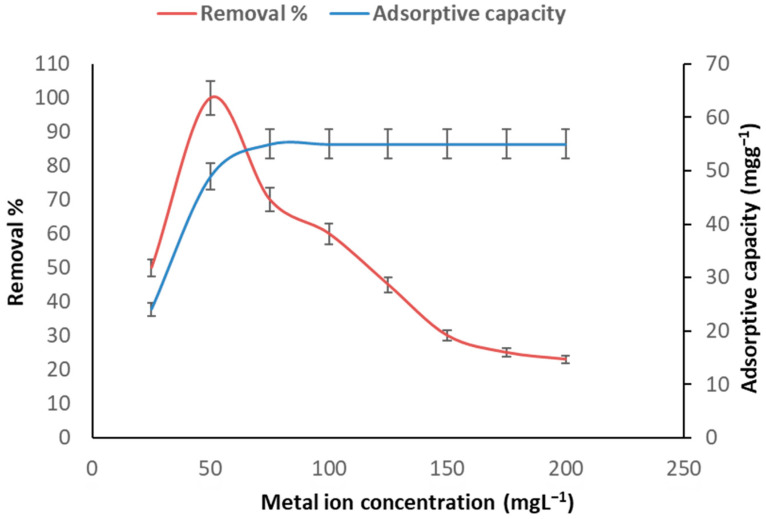
As (III) bio-adsorption percentages at various metal ion concentrations. The biomass dose was 1 gL^−1^, the pH was 6.0, and the contact period was 76 min.

We evaluated the highest concentration of arsenic ions that could be adsorbed by the algal absorbent at a 1 g bio-adsorbent dose with pH of 6.0 and a contact time of 76 min. Figure 4 displays the arsenic bio-adsorption rate by *C. vulgaris* at different concentrations of arsenic ions. The bio-adsorption capacity of As (III) grew proportionally with the initial concentration, registering a 50 mgL^−1^ bio-adsorption capacity with a 55 mgg^−1^ removal percentage of 100%. The minimum and maximum As (III) bio-adsorptive capacities were 24 mgg^−1^ and 55 mgg^−1^, respectively, with As (III) concentrations of 25 and 50 mgL^−1^. An increase in the initial As (III) concentration may have resulted in inconsistent metal ions being in contact with the algal absorbent surface, which could have led to mass transfer resistance between the aqueous and absorbent phases. Nonetheless, increasing the As (III) ion concentration from 50 to 200 mgL^−1^ did not increase the rate of uptake capacity, with the capacity remaining constant at 55 mgg^−1^. This indicated that near-constant saturation was achieved and that there were limited binding sites available for adsorption [17].

### 3.2. Elemental Analysis of Algal Adsorbent

The percentages of the most critical element on the algal adsorbent surface (Table 1) were oxygen (39.87%), carbon (43.65%), hydrogen (8.51%), nitrogen (6.98%), and sulfur (1.11%), which formed the functional groups (carboxyl, amines, alcohol, phenol, etc.).

### 3.3. Scanning Electron Microscopy

**Figure 5:**
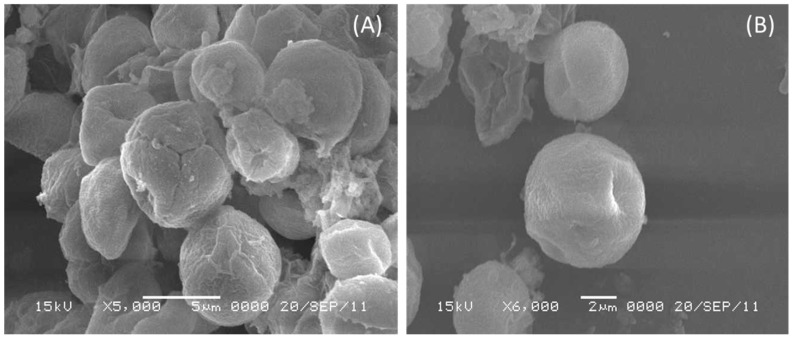
SEM micrographs of (**A**) arsenic-untreated biomass as a blank sample and (**B**) arsenic-adsorbed biomass.

Figure 5A,B demonstrate the surface morphology of blank and arsenic-treated algal cells. The prevalence of a heterogeneous porous structure and its preceding structures with coarse holes, which were significant for the prospective adsorbent, can be noticed in the blank picture (Figure 5A). As (III) confinement caused the treated algal cells (Figure 5B) to assume a tight and smooth structure throughout the image [40].

### 3.4. FTIR Analysis

**Figure 6:**
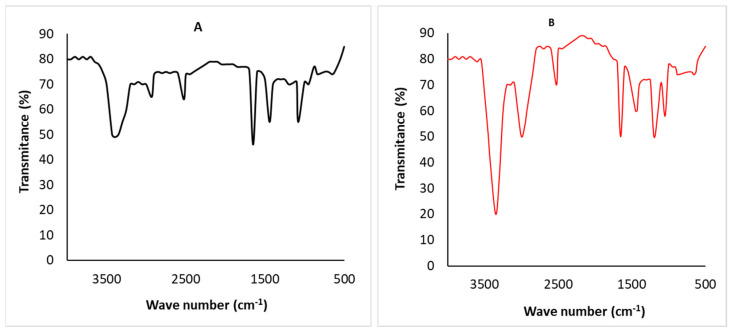
FTIR spectra of (**A**) untreated arsenic biomass as a blank sample and (**B**) arsenic-treated biomass.

FTIR spectroscopy was used to identify several functional groups present on the surface of the algae. The FTIR spectra of the algal adsorbent revealed several types of vibrational frequencies due to distinct functional groups. The FTIR graphs of the original and chemically transformed algal samples are shown in Figure 6A,B. The FTIR spectroscopic graph revealed an intensity peak at 3300 cm^−1^, indicating the existence of O-H and N-H [41]. According to Gupta et al. [42], a prolonged rise was seen to be displaced in a treated algal biomass compared with an untreated algal biomass. Peaks at 3000 cm^−1^ correlated with the C-H stretching vibrations in cellulose and lignin [43]. Peaks near 1653 cm^−1^ were also linked to C=O asymmetric stretching. More significant shifting revealed a tight connection between the metal ions (As (III)) and the algal surface. Plazinski et al. [44] reported that the peak at 1200.10 cm^−1^ was due to -SO_3_ with aliphatic amine stretching (C-N). Compared with unmodified *C. vulgaris*, the strength of the O-H, C-H, and C=O stretching bands for modified *C. vulgaris* increased, indicating the increase in these functional groups. The FTIR graph showed that the *C. vulgaris* surface was occupied by the hydroxyl, carboxylate, amino, and amide groups, which had a remarkable role during the adsorption process [45].

The mechanisms of adsorption can be generally categorized as physisorption and chemisorption. The physisorption mechanism involves adsorption between the surface and adsorbate, which includes a range of factors such as diffusion, van der Waals interactions, hydrogen bonding, electrostatic interactions, and exothermal phenomena [46]. On the other hand, the chemisorption mechanism involves electronics, valence forces that prevail between the absorbent and adsorbate, chemical bonding to the adsorbent’s surface, complex formation, chelation, proton displacement, redox reaction, and covalent bonding [47].

### 3.5. Bio-Adsorption Isotherm

The bio-adsorption isotherm at a given condition represents the equilibrium relationship between the adsorbate concentration in an aqueous solution and that on the adsorbent’s surface of microalgae *C. vulgaris*. Langmuir, Freundlich, and Dubinin–Radushkevich models were used to describe the equilibrium relationships. Table 2 depicts the equilibrium adsorption data and summarized isotherm models. According to the regression coefficients, the Langmuir isotherm model fitted the experimental equilibrium data better than the Freundlich isotherm and Dubinin–Radushkevich isotherm models. The As (III) bio-adsorption data (R^2^; As = 0.9894) suited the Langmuir isotherm model better for *C. vulgaris* than the Freundlich and Dubinin–Radushkevich isotherm models (R^2^; As = 0.7227 and 0.951, respectively). The Langmuir maximum bio-adsorption capacity (q_max_) was 45 mgg^−1^ whilst in the case of the Dubinin–Radushkevich model, it was 8.7 mgg^−1^. The As (III) adsorption behavior approximated the Langmuir isotherm better (R^2^; As = 0.986), implying that the nature of adsorption was homogeneous via monolayer adsorption [48,49,50]. This suggested that *C. vulgaris* could successfully remove As (III) from aqueous solutions.

### 3.6. Bio-Adsorption Kinetics

**Figure 7:**
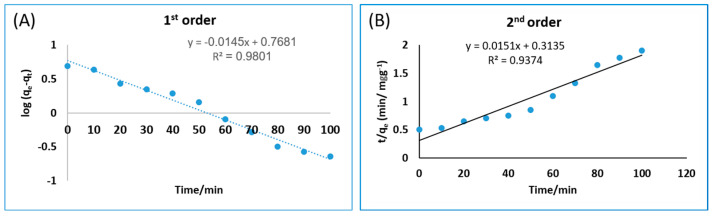
(**A**) Pseudo-first-order and (**B**) pseudo-second-order kinetics of As (III) bio-adsorption at pH 6, 50 mgL^−1^ arsenic, 1 gL^−1^ bio-adsorbent, and 1 g bio-adsorbent.

Experiment data were evaluated with pseudo-first-order and pseudo-second-order models to investigate the As (III) bio-adsorption kinetics on *C. vulgaris*. The values of the constant kinetic parameters for As (III) bio-adsorption are shown in Table 3. The R^2^ for As (III) calculated from the pseudo-first-order model was significantly higher (0.9801) than the values calculated from the pseudo-second-order kinetic model for *C. vulgaris*. The observed qe values (3.130 mgg^−1^) were confirmed by the computed qe values for As (III), derived using the pseudo-second-order model with the adsorbent [33]. Figure 7A shows a linear plot of As (III) and the correlation coefficient values (R^2^ = 0.9801 for an initial concentration of 50 mgL^−1^). This indicated that the bio-adsorption of arsenic ions was best through pseudo-first-order kinetics. The slope and intercept of the plot in Figure 7B were used to calculate the bio-adsorption parameters q_e_ and k_2_.

### 3.7. Thermodynamics of As (III) Bio-Adsorption

The temperature parameters during the sorption process are of immense significance. An increase in temperature generally accelerates the rate of chemical reactions. As part of the investigation, the Van’ t Hoff equation and other relations were used to calculate the activation energy of arsenic sorption. Table 4 shows the values of ΔH = 31.214 kJ mol^−1^, ΔS  =  104.320 kJ mol^−1^ K^−1^, and ΔG  =  −1.76 to −4.98 kJ mol^−1^ K^−1^. Endothermic adsorption was indicated by the positive value of ΔH, whilst the negative value of ΔG implied spontaneous adsorption. The low activation energies of arsenate bio-adsorption on *C. vulgaris* implied that the sorption of arsenic on the algae could involve both physical and activated processes. However, the positive heat of adsorption of the bio-sorbent indicated that endothermic adsorption occurred [51]. Based on the experimental findings, increasing the temperature raised the arsenic bio-adsorption on *C. vulgaris*, indicating an endothermic process. Moreover, all free energy values at various temperatures were negative, indicating that arsenic adsorption on the adsorbents evaluated occurred spontaneously.

### 3.8. Comparison between C. vulgaris (SAG 211-11b) with Other Adsorbents for Bio-Adsorption of Arsenic (III)

Table 5 compares the maximum bio-adsorption capacities (qmax) of arsenic (III) by various adsorbents tested in the literature with *C. Vulgaris* (SAG 211-11b). It demonstrates that the qmax values for various adsorbents vary significantly. In this study, *C. vulgaris* (SAG 211-11b) (20.9 mgg^−1^ at pH 6) had a potential adsorption efficiency for arsenic (III) from aqueous solutions when compared with *Chlamydomonas* sp. (53.8 mgg^−1^ at pH 4), *C. vulgaris* (13 mgg^−1^ at pH 6), *C. vulgaris* modified with NaCl (20.9 mgg^−1^ at pH 6), and *Spirulina platensis* modified with ZnCl2 NaCl (24.8 mgg^−1^ at pH 6). The results in Table 5 show that *C. vulgaris* (SAG 211-11b) as a bio-adsorbent was the best in the bio-adsorption of arsenic (III) from aqueous environments.

## 4. Conclusions

The study aimed to investigate the ability of green algae *C. vulgaris* to bio-adsorb As (III) simultaneously. To achieve the bio-adsorption of As (III) ions from aqueous solutions, *C. vulgaris* was employed. The findings indicated that the adsorption rate and removal percentage of As (III) ions were subject to the pH, initial metal concentrations, biomass dosage, and contact time as well as a rougher surface structure and larger specific surface area of bio-adsorbents. The optimal adsorptive ability of As (III) was found to be 55 mgg^−1^ with a 100% removal percentage. Among the models used, Langmuir was found to be the most precise model for As (III) bio-adsorption on the bio-adsorbent at an uptake of 45 mgg^−1^ compared with the Freundlich and Dubinin–Radushkevich isotherm models. The research concluded that the bio-adsorption process was endothermic and spontaneous, as per the thermodynamic studies. Consequently, the study proposed an efficient and environmentally friendly solution to simultaneously eliminate As (III) by *C. vulgaris* (SAG 211-11b).

## Figures and Tables

**Table 1:** The proportion of elemental compositions in an algal adsorbent.

Element	Oxygen	Carbon	Hydrogen	Nitrogen	Sulfur
Percentage	39.87	43.65	8.51	6.98	1.11

**Table 2:** Langmuir, Freundlich, and Dubinin–Radushkevich isotherm data of As (III) bio-adsorption on algal adsorbents at pH 6, arsenic concentrations ranging from 50 to 200 mgL^−1^, 1 gL^−1^ adsorbent dose, and 76 min incubation.

Langmuir Isotherm		
q_max_ (mgg^−1^)	K_L_ (Lmg^−1^)	R^2^
45	0.21	0.9894
Freundlich Isotherm		
K_f_ (Lg^−1^)	n	R^2^
1.44	1.459	0.7227
Dubinin–Radushkevich Isotherm		
q_D–R_ (mgg^−1^)	B_D–R_	R^2^
8.7	0.002	0.951

**Table 3:** Arsenic ion (III) bio-adsorption kinetics parameters of *C. vulgaris* as a bio-adsorbent.

Parameter	Pseudo-First-Order			Pseudo-Second-Order		
***C.*** *vulgaris* biosorbent	K_1_	q_e_	R^2^	K_2_	q_e_	R^2^
	8.78 × 10^−5^	3.130	0.9801	2.29 × 10^−4^	0.795	0.9374

**Table 4:** Thermodynamic parameters related to As (III) bio-adsorption by *C. vulgaris*.

ΔH (kJ mol^−1^)	ΔS (kJ mol^−1^ K^−1^)	ΔG (kJ mol^−1^ K^−1^)		
		289 K	308 K	318 K
31.214	104.320	1.76	3.43	4.98

**Table 5:** A comparison of the arsenic adsorption capacities of *C. vulgaris* and other adsorbents.

Absorbates	pH	qmax (mgg^−1^)	References
Mg0.27Fe_2_·5O_4_	7	83.2	[52]
Fe_3_O_4_-GO (MGO)	6.5	59.6	[53]
FeMnOx/RGO	7	22.22	[53]
CeO_2_–graphene composite	4	1.019	[54]
GO-ZrO(OH)_2_	5–11	84.89	[55]
nZVI/graphene	7	29	[56]
Magnetic graphene	4	3.26	[57]
Fe_3_O_4_/graphene/LDH	6	73.1	[57]
Magnetic GO	4	38	[58]
Magnetic rGO	4	12	[59]
MnFe_2_O_4_	3	94	[60]
CoFe_2_O_4_	3	74	[60]
CuFe_2_O_4_ order to further	7	82.7	[61]
GNP_s_/Fe-Mg oxide	7	103.9	[62]
GNP_s_/CuFe_2_O_4_	4	172.7	[62]
*Chlamydomonas* sp.	4	53.8	[63]
*C. vulgaris*	6	13	[64]
*C. vulgaris* modified with NaCl	6	20.9	[65]
*Spirulina platensis* modified with ZnCl_2_	6	24.8	[66]
*C. vulgaris* (SAG 211-11b)	6	55	This work

## Data Availability

The datasets obtained and analyzed in the current study are available from the corresponding author on reasonable request.
